# Prevalence of Antimicrobial-resistant Bacteria in HACCP Facilities

**DOI:** 10.14252/foodsafetyfscj.D-23-00004

**Published:** 2023-09-22

**Authors:** Ramesh Subramaniam, Nuzul Noorahya Jambari, Kuan Chee Hao, Ungku Fatimah Ungku Zainal Abidin, Nor Khaizura Mahmud, Ab Rashid

**Affiliations:** 1 Chemsil Air & Water Sdn Bhd, 33, Jalan Kota Raja H27/H, Hicom Town Centre, Seksyen 27, 40400, Shah Alam, Selangor, Malaysia; 2 Department of Food Science, Faculty of Food Science and Technology, Universiti Putra Malaysia, 43400 UPM Serdang, Selangor, Malaysia; 3 Laboratory of Food Safety and Food Integrity, Institute of Tropical Agriculture and Food Security, Universiti Putra Malaysia, 43400 UPM Serdang, Selangor, Malaysia; 4 Department of Food Service and Management, Faculty of Food Science and Technology, Universiti Putra Malaysia, 43400 UPM Serdang, Selangor, Malaysia; 5 Huru Biotech Sdn Bhd, No.1 Jalan Perindustrian Balakong Jaya, 2/2, Taman Perindustrian Balakong Jaya, 2, 43300 Balakong Jaya, Selangor, Malaysia

## Abstract

Foodborne pathogens, such as *Staphylococcus aureus* and *Salmonella* spp., develop antimicrobial resistance (AMR) over time, resulting in compromised food safety. Therefore, this study aimed to determine the prevalence, compliance against Malaysia’s veterinary standing procedure directive (APTVM 16 (c): 1/2011): Appendix 7^13^^)^, and antimicrobial resistance (AMR) profiles of *S. aureus* and *Salmonella* spp., in raw poultry meat, poultry meat products, and poultry-based ready-to-eat (RTE) foods. Here, 699 raw poultry meat and meat products samples were obtained from selected hazard analysis critical control points (HACCP)-certified poultry meat-processing plants. Additionally, 377 samples of poultry-based RTE meals were collected from dine-in establishments and hospital catering facilities in Klang Valley, Malaysia. *Salmonella* spp. and *S. aureus* were present in 2.1% and 2.8% of the analyzed samples, respectively. *Salmonella* spp isolated from raw poultry meat and its products displayed resistance to ampicillin (100%), chloramphenicol (87.0%), cefuroxime (60.9%), cefazolin (56.5%), and kanamycin (52.2%). Similarly, *S. aureus* isolated from raw poultry meat, its products, and poultry-based RTE foods exhibited resistance against tetracycline, chloramphenicol, penicillin, ciprofloxacin, trimethoprim, kanamycin, and cefoxitin. The multi-antibiotic resistance (MAR) demonstrated by these foodborne pathogens makes their prevalence disconcerting. This highlights the need for more stringent monitoring and enduring sanitary and hygiene practices in HACCP establishments to prevent foodborne infections and potential transmission of AMR bacteria.

## Introduction

The discovery of the effectiveness of antimicrobials as growth promoters in the mid-1950s led to their prevalent use in the poultry industry. Antimicrobials are used at sub-therapeutic doses to enhance the feed-to-weight ratio and protect animals against common pathogens. However, foodborne pathogens develop antimicrobial resistance (AMR) owing to selective pressure^1^^)^. This subsequently jeopardizes food safety^2^^,^^3^^,^^4^^)^.

*Staphylococcus aureus* and *Salmonella* spp. are two of the most common foodborne bacteria associated with poultry meat^5^^,^^6^^,^^7^^)^. They are linked to numerous outbreaks of foodborne illness^8^^)^ owing to their antimicrobial resistance^9^^)^. Although these AMR bacteria typically result in a mild self-limiting illness, they can cause severe symptoms in immunocompromised people. Hence, the European Centre for Disease Prevention and Control (ECDC)^10^^)^ has classified *Salmonella* spp. as one of the most prevalent foodborne zoonotic pathogens in Europe.

In response to a call by the world health organization (WHO) to combat antibiotic resistance worldwide^11^^)^. Malaysia released a 5-year AMR action plan in 2017^12^^)^. However, the prevalence and patterns of AMR in *S. aureus* and *Salmonella* spp. are not well documented in processed poultry meat and poultry-based ready-to-eat (RTE) foods prepared and served at hazard analysis critical control point (HACCP) establishments. HACCP certifications are provided by the Ministry of Health and third-party certification bodies such as SIRIM QAS International and Bureau Veritas in Malaysia.

This gap in extant knowledge is addressed in this study. We analyzed samples from selected HACCP-certified poultry meat processing plants, as well as RTE poultry-based meals served in HACCP-certified food outlets and hospitals in Klang Valley, Malaysia, to determine the prevalence, compliance with Malaysia veterinary standing procedure directive APTVM 16 (c): Appendix 7^13^^)^, and AMR profile in *S. aureus* and *Salmonella* spp.

## Material and Methods

### Sample Collection

Between 2018 and 2020, we analyzed 699 poultry meat and its products, and 377 poultry-based RTE foods (Table 1). Samples were collected using a non-probability convenience sampling method. All collected samples were aseptically placed in a sterile bag maintained at 0–4°C, transported to the laboratory, and analyzed within 24 h.

**Table 1.** Prevalence, range, and reference limits of *S. aureus* and *Salmonella* spp. isolated from selected HACCP-certified establishments.

Sample Type	*S. aureus* (MPN/g)			*Salmonella*.spp Absent/present	
	Prevalence (%) n	Range	Reference limits*	Prevalence (%) n	Reference limits*
Raw and semi cooked meat products					
Raw poultry meat from three meat processing establishments (carcass, skinless, boneless meat, raw cut, by-products)	(2.9) 7/241	3.6 - 240	250	(6.2) 15/241	Absent
Processed poultry meat product from three meat processing establishments: (burgers, frankfurters, marinated fillets, nuggets, and minced meat).	(2.0) 9/458	3.6 - 11	250	(1.7) 8/458	Absent
Total	(2.3) 16/699			(3.3) 23/699	
Meat and meat product ready for consumption					
Poultry-based RTE food from 16 dine-in establishments (hotels, shopping malls, and franchised food outlets)	(3.5) 9/254	11 - 63	Absent	Absent	Absent
Poultry-based RTE food from four hospital facilities (inpatient food and cafeteria)	(4.1) 5/123	20 - 40	Absent	Absent	Absent
Total	(3.7) 14/377			Absent	
Overall Total	(2.8) 30/1076			(2.1) 23/1076	

RTE, ready-to-eat food; HACCP, Hazard analysis and critical control point; n, number of samples; MPN: Most Probable Number; Reference limits*, Malaysia Veterinary Document Register (APTVM 16 (c):1/2011): Appendix 7

### Isolation of *S. aureus*

The most probable number (MPN) test method was adopted according to AOAC 987.09 (Association of Official Analytical Chemists) to detect and quantify *S. aureus*. Briefly, 50 g of aseptically homogenized sample was added to 450 mL of 0.1% buffered peptone water (BPW; Merck, Germany) to obtain 1:10 dilution, which was diluted further by transferring 10 mL of the prepared volume to 90 mL of dilution water to achieve serial dilution of 10^-2^ to 10^-6^. Subsequently, 1 mL, 0.1 mL, and 0.01 mL of aliquots were transferred to tubes containing tryptic soy broth (TSB; Merck, Germany), 10% sodium chloride, 1% sodium pyruvate (SA Broth; Merck, Germany), in triplicate. All tubes were incubated for 48 ± 2 h at 35°C. Tubes with growth or turbid content were mixed, and one loopful was transferred onto dried Baird-Parker agar plates (BP; Merck, Germany) and were incubated for 48 h at 35–37°C. A well-isolated colony from each growth plate was confirmed by a bactident coagulase test using brain heart infusion (BHI; Merck, Germany) broth and rabbit plasma with EDTA. Any degree of clot formation indicates the presence of coagulase-positive *S. aureus*. 

### Isolation of *Salmonella* spp.

To carry out non-selective enrichment, 25 g of the sample was transferred into a container with 225 mL BPW (Merck) and incubated at 37 ± 1°C for 16-20 h. Next 1 mL of the sample was inoculated into 10 mL of Muller-Kauffmann tetrathionate-novobiocin broth (MKTTn; Merck,) and incubated at 37 ± 1°C for 24 ± 3 h to carry out selective enrichment. Subsequently, 0.1 mL of the same sample was placed into 10 mL of Rappaport Vasiliadis Soy (RVS; Merck) broth and incubated at 41.5 ± 1°C for 24 ± 3 h. Incubated MKTTn and RVS broth were streaked on xylose lysine deoxycholate agar (Merck) and Brilliance Salmonella agar (Oxoid, England) and further incubated at 37 ± 1°C for 24 ± 3 h and 36 ± 1°C for 48 h, respectively. The suspected *Salmonella* spp. was confirmed biochemically using Microgen GN-ID A+B system in accordance with the manufacturer’s^14^^)^ instruction (Microgen Bioproducts, Camberley, UK).

### Antimicrobial Susceptibility Test

The antimicrobial susceptibility of all samples was determined using the Kirby-Bauer standard disk diffusion method on Mueller-Hinton agar (Oxoid, England). *Salmonella* spp. and *S. aureus* were tested with 12 and seven antimicrobials from seven and six classes, respectively. Antimicrobial discs (Liofilchem, Italy) were placed on the agar surface with an overnight culture that was prepared and incubated at 37 °C for 18−24 h. Based on the size of the inhibition zones formed, the specimens were rated as sensitive or resistant in accordance with the CLSI (27th)^15^^)^ and EUCAST (2017)^16^^)^ guidelines. *E. coli* ATCC 25922 and *S. aureus* ATCC 25923 were used as quality control strains.

## Results and Discussion

While the prevalence of *Salmonella* spp. and *S. aureus* obtained in this study (Table 1) is lower than that reported for poultry meat in Cambodia^17^^)^, Egypt^18^^)^, India^19^^)^, Morocco^20^^)^, and Thailand^21^^)^ the noncompliance of *Salmonella* spp. in poultry meat processing plants requires attention. In HACCP-certified poultry processing plants in Nepal^22^^)^, *S. aureus* was more prevalent than in our study, whereas *Salmonella* spp. was not detected. The absence of *Salmonella* spp. in RTE foods indicates a considerable improvement in the microbiological quality in HACCP dine-in establishments. In contrast, the prevalence and noncompliance of *S. aureus* were higher in RTE foods than in raw poultry meat. The prevalence of *S. aureus* strains in RTE samples obtained from dine-in establishments and hospital catering facilities was comparable to that reported in China (4.3%: retail food)^23^^)^. However, this was lower than the 50.0% observed in cooked food samples obtained from food premises in Klang Valley^24^^)^. The higher prevalence of *S. aureus* and noncompliance in poultry-based RTE foods compared to raw meat and its product is likely due to unhygienic food handling practices or cross-contamination, indicating a further improvement in sanitization and hygiene practices is required.

The administration of antimicrobials to chicken at therapeutic and subtherapeutic levels has been an integral part of poultry meat production for decades^25^^,^^26^^,^^27^^)^. Thus, the presence of MAR *Salmonella* spp. (Table 2) and *S. aureus* (Table 3,4) was expected. The AMR in *Salmonella* spp. and *S. aureus* - observed in this study were consistent with findings obtained in other countries^6^^,^^26^^)^, indicating their prevalence and the need for their use to be controlled^28^^)^. Moreover, the high MAR index values obtained in this study suggest that the isolated bacteria can cause infections that are difficult to treat.

**Table 2.** Antimicrobial resistance profile against all antimicrobial tested and multiple resistance index (MAR) of *Salmonella* spp. isolated from poultry meat processing plants.

**Antimicrobials** n (%)											
AMP ß-Lactam	CRO ß-Lactam	CAZ ß-Lactam	TE Tetracycline	CIP Fluoroquinolones	NA Quinolone	TM Sulfonamides	C Phenicol	K Aminoglycosides	KZ ß-Lactam	FOX ß-Lactam	CXM ß-Lactam
23 (100)	8 (34.8)	11 (47.8)	3 (13.0)	5 (21.7)	5 (21.7)	10 (43.5)	20 (87.0)	12 (52.2)	13 (56.5)	5 (21.7)	14 (60.9)
Number of antimicrobials resistance isolates				MAR Index (*a/b*)				Antimicrobial Resistance Profile (n)			
2				(0.17)				AMP-NA (1), AMP-KZ (2)			
3				(0.25)				AMP-C-KZ (1)			
4				(0.33)				AMP-TM-C-CXM (2), AMP-TM-C-KZ (2)			
5				(0.42)				AMP-NA-C-K-KZ (1), AMP-CRO-CAZ-NA-C (1)			
6				(0.50)				AMP-CRO-CAZ-NA-C-CXM (1), AMP-CAZ-CIP-TM-C-KZ (1)			
7				(0.58)				AMP-CIP-TM-C-K-KZ-CXM (1), AMP-CRO-CAZ-NA-C-K-CXM (2),AMP-TM-C-K-KZ-FOX-CXM (1)			
8				(0.67)				AMP-CIP-TM-C-K-KZ-FOX-CXM (1), AMP-CAZ-TE-NA-C-K-FOX-CXM (1), AMP-CAZ-NA-TM-C-K-KZ-CXM (1), AMP-CRO-CAZ-TE-C-K-KZ-CXM(1)			
9				(0.75)				AMP-CRO-CAZ-CIP-NA-C-K-KZ-CXM (1),AMP-CRO-CAZ-TE-NA-C-K-FOX-CXM (1)			
10				(0.83)				AMP-CRO-CAZ-CIP-NA-TM-C-K-FOX-CXM (1)			

Abbreviation: n, number of isolates; MAR, multiple antibiotic resistance; a, number of resistance antimicrobial; b, total number of antimicrobials used; AMP, ampicillin (10 μg); CRO, ceftriaxone (30 μg); CAZ, ceftazidime (30 μg); TE, tetracycline (30 μg); CIP, ciprofloxacin (5 μg); NA, nalidixic Acid (30 μg); TM, trimethoprim (5 μg); C, chloramphenicol (30 μg); K, kanamycin (30 μg); KZ, cefazolin (30 μg); FOX, cefoxitin (30 μg); CXM, cefuroxime (30 μg)

**Table 3.** Antimicrobial resistance profile of *S. aureus* isolates from raw poultry meat and processed poultry meat products.

**Antimicrobials** n (%)								
TE Tetracycline	CIP Fluoroquinolones		C Phenicol		K Aminoglycosides	P ß-Lactam	FOX ß-Lactam	TM Sulphonamides
12 (85.7)	4 (28.6)		11 (78.6)		6 (42.9)	10 (71.4)	2 (14.3)	5 (35.7)
Number of antimicrobials resistance isolates		MAR Index (*a/b*)		Antimicrobial Resistance Profile (n)				
2		(0.29)		TE-C (1), CIP-C (1), TE-K (1)				
3		(0.43)		TE-C-P (2), CIP-P=TM (1)				
4		(0.57)		TE-C-P-TM (3), TE-C-K-P (1), TE-CIP-C-L (1)				
5		(0.71)		TE-CIP-K-P-FOX (1), TE-C-K-P-FOX (1), CIP-C-K-P-TM (1)				

Abbreviations: n, number of isolates; MAR, multiple antibiotic resistance; a, number of resistance antimicrobial; b, total number of antimicrobials used; TE, tetracycline (30 μg); CIP, ciprofloxacin (5 μg); C, chloramphenicol (30 μg); K, kanamycin (30 μg); P, penicillin (10 μg); FOX, cefoxitin (30 μg); TM, trimethoprim (5 μg)

Other Malaysian researchers have reported 24.0%, 100%, and 47.7% *Salmonella* spp. ampicillin resistance in poultry breeding and processing environments^29^^)^, in poultry farms^30^^)^, and in broiler farms^27^^)^ respectively. A similar *Salmonella* spp. to ampicillin resistance (96.0%) was reported in a study conducted on Nigerian poultry farms^31^^)^. In contrast, lower resistance (13.6%) to chloramphenicol than that observed in our study was reported in a global overview of poultry meat by Castro-Vargas et al^7^^)^. Moreover, 62.2% and 44.4% resistance were reported by Hamed et al^32^^)^ (poultry farm) and Shehata et al^33^^)^ (poultry hatcheries and broiler chickens), respectively, in Egypt. Similarly, Eguale^34^^)^ noted 42.3% resistance in Ethiopian poultry farms.

All *S. aureus* isolates from RTE food were resistant to at least one antimicrobial (Table 4), which was comparable to the 98.4% reported by Xing et al^35^^)^. Isolated *S. aureus* strains were highly resistant to tetracycline, chloramphenicol, and penicillin. The percentage of penicillin-resistant isolates in this study was higher than that reported for South African^6^^)^ poultry meat and its products (55.8%). *S. aureus* isolated were also highly resistant to ciprofloxacin (68.8%), compared to 50.0% (nasal swab: poultry farm) and 17.6% (nasopharyngeal swab: slaughterhouse) reported in Algeria^36^^)^ and Morocco^37^^)^, respectively. These show the prevalent use of these antibiotics as anti-inflammatory agents^38^^)^ and growth promoters^39^^)^ in livestock. Here MRSA prevalence was determined based on the resistance of *S. aureus* to cefoxitin, which was observed in 31.2% of the analyzed samples. Lower MRSA resistance rates were reported in Thailand^21^^)^ (7.89%: poultry meat), Tehran^40^^)^ (7.62%: raw and cooked hospital food), South Africa^41^^)^ (20.8%: poultry products), and African countries^42^^)^ (9%: meat and meat products). A higher prevalence was observed in South Africa^43^^)^ (91.7%: poultry), and Germany^44^^)^ (37.2%: poultry). This wide variation in MRSA resistance rates between countries is likely due to varying antimicrobial usage regulations and guidelines in poultry production.

**Table 4.** Antimicrobial susceptibility of 14 Coagulase-positive *S. aureus* isolates from RTE poultry-based meals served at dine-in and hospital catering facilities based on the results of the disk diffusion method.

**Antimicrobials** n (%)								
TE Tetracycline	CIP Fluoroquinolones		C Phenicol		K Aminoglycosides	P ß-Lactam	FOX ß-Lactam	TM Sulphonamides
14 (87.5)	11 (68.8)		13 (81.2)		7 (43.8)	13 (81.2)	5 (31.2)	8 (50.0)
Number of antimicrobials resistance isolates		MAR Index (*a/b*)		Antimicrobial Resistance Profile (n)				
2		(0.29)		TE-K (1)				
4		(0.57)		TE-C-K-P (1), TE-CIP-C-P (1), TE-CIP-K-FOX (1), TE-C-P-TM (3), TE-CIP-C-P (4)				
5		(0.71)		TE-CIP-P-FOX-TM (1), CIP-C-K-FOX-TM (1), CIP-C-K-P-TM (2), TE-CIP-C-K-FOX (1)				

Abbreviations: n, number of isolates; MAR, multiple antibiotic resistance; a, number of resistance antimicrobial; b, total number of antimicrobials used; TE, tetracycline (30 μg); CIP, ciprofloxacin (5 μg); C, chloramphenicol (30 μg); K, kanamycin (30 μg); P, penicillin (10 μg); FOX, cefoxitin (30 μg); TM, trimethoprim (5 μg)

AMR imposes a significant economic burden on the hospitality sector owing to the emergence of novel strains and growing resistance to most available antibiotics^36^^,^^45^^)^. These studies provide evidence for the application of HACCP plans to systematically identify and minimize the spread of MRSA and AMR pathogens during poultry meat processing, distribution, and subsequently, food preparation for consumption. However, new approaches are needed to address these health threats, such as the “One Health” initiative aimed at managing infectious disease outbreaks while promoting food safety and security^19^^,^^46^^)^. As this study was limited to the Klang Valley region in Malaysia, the results can only be generalized to further studies conducted in HACCP-certified establishments nationwide and in different settings.

This study also demonstrates that AMR is prevalent in *Salmonella* spp. and *S. aureus* isolated from poultry meat samples obtained from Malaysian poultry meat processing plants and food serving establishments. Thus, the effort to improve HACCP plans toward the reduction of contamination with AMR pathogens, inhibiting their growth and subsequently eliminating them, is highly recommended.

## CRediT Authorship Contribution Statement

Ramesh Subramaniam: Conceptualization, Data curation, Formal analysis, Methodology, Writing – original draft. Nuzul Noorahya Jambari: Writing – review & editing. Kuan Chee Hao: Writing – review & editing. Nor Khaizura Mahmud Ab Rashid: Writing – review & editing. Ungku Fatimah Ungku Zainal Abidin: Writing – review & editing.
